# Nutraceutical profiling of elite onion germplasm and breeding hybrids with improved nutraceutical quality

**DOI:** 10.1371/journal.pone.0262705

**Published:** 2022-01-19

**Authors:** Najma Tabussam, Rashid Mehmood Rana, Muhammad Kausar Nawaz Shah, Muhammad Sheeraz Ahmad, Muhammad Sajjad, Yongqiang Lu

**Affiliations:** 1 Department of Plant Breeding and Genetics, Pir Mehr Ali Shah Arid Agriculture University, Rawalpindi, Pakistan; 2 Department of Biochemistry, Pir Mehr Ali Shah Arid Agriculture University, Rawalpindi, Pakistan; 3 Department of Biosciences, COMSATS University Islamabad (CUI), Islamabad, Pakistan; 4 Yunnan Academy of Agricultural Sciences, Kunming, Yunnan Province, P.R. China; Government College University Faisalabad, PAKISTAN

## Abstract

Onion (*Allium cepa* L) is a major reservoir of important nutraceutical ingredients. Herein, nutraceutical profiling of elite germplasm was assessed and hybrids with improved nutraceutical quality were selected. The nutraceutical components were screened through Fourier Transform Infrared Spectroscopy (FTIR) analysis (scan range 4000-400cm^-1^) followed by spectrophotometric/colorimetric quantification in oven dried bulb samples. Line × Tester (L×T) analysis was used to identify potential hybrids with better nutraceutical quality. Based on common functional groups obtained from FTIR analysis, as well as bulb color, the onion genotypes were categorized into six groups viz., white, yellowish brown, light brown, dark brown, brown and purplish brown. Results indicated that the purplish brown, yellowish brown and dark brown genotypes had maximum concentration of pyruvic acid, total flavonoids and total phenolic content, while vitamin C content showed weak association with color pigmentation. The onion variety ‘Onion Swat’ contained the highest level of pyruvic acid (17.18 μM) and ‘MKS8823GO’ had the highest vitamin C content (13.83mg/100mL). The L×T analysis revealed that out of 35 crosses, ‘MKS-77127 × Onion Swat’ and ‘MKS-77127 × MKS777’ were the best hybrids with improved nutraceutical quality. Further, observations for specific combining ability, general combining ability, genetic versus environmental variance, heritability and heterosis indicated that the studied parameters were genetically inherited and could be improved significantly by adopting an appropriate breeding strategy.

## Introduction

Vegetables are important since they contain components of nutritional importance including vitamins, minerals, phytonutrients and fibre as well as provide some carbohydrates. Vegetables are rich in antioxidants conferring defence against chronic diseases such as diabetes, cancer, obesity, syndrome and cardiovascular diseases [1]. Amongst vegetables, onion occupies a central position being a source of number of phytonutrients that are reputed for nutritional and pharmaceutical value [2].

Onion is recognized as a plant of medicinal importance and considered to mitigate the effect of serious risks associated with various diseases [3]. Onion genotypes may be sweet or pungent. Onion has various sulfur containing compounds that cause pungency through a series of reactions. When an onion tissue is raptured the aliinase enzyme comes into contact with flavor precursors, S-alk(en)yl-cysteine–sulfoxides and several volatile compounds, pyruvic acid and ammonia are produced during the aliinase reaction. Thus, amount of pyruvic acid produced during this reaction is best indicator of flavor precursors and has been used to measure onion pungency [4]. Phytochemicals such as flavonoids, phenolics and anthocyanins present in onion have therapeutical and pharmaceutical utility [5]. Phytochemicals presence in onion justifies various health benefits associated with onion, for example, it reduces the risk of gastric ulcer by scavenging free oxygen radicals and also by inhibiting the development of ulcer forming microorganism. It is also used as an antibacterial and antiparasitic agent [6].

Genetic diversity assessment is of prime importance for sustainable crop improvement through breeding programs [7]. The hybrid performance is reported superior to open pollinated varieties with respect to yield and nutritional quality traits [8]. FTIR spectroscopy is used to determine antioxidant potential of vegetables due to its rapid and precise estimation. It is also used to grouping of genotypes for different levels of nutraceutical components [9].

Genetics of nutraceutical quality in onion is still not explored due to attention being focused mainly on horticultural traits including disease resistance, storability, growth yield, and bolting etc. [10]. Phenolics and flavonoids are major classes of phytochemicals which impart color, taste and texture. These components are high in red and yellow onions and low in white onions. The flavonoids and phenolics contents vary with color of onion bulb, variety and type of onion [11–16].

Due to range of health benefits associated with these nutraceutical components and consumer’s preferences for better quality onion has increased demand for improved onion cultivars. To identify onion hybrids with better nutraceutical quality, a 5×7 L×T mating design was used. The results provide a reference for the commercial production of the identified onion hybrids with better nutraceutical quality.

## Materials & methods

### Collection of seeds

Seeds of 39 local and exotic onion genotypes (S1 Table) were obtained from National Agriculture Research Centre (NARC) Islamabad, Pakistan and Magnus Kahl Seeds (MKS), Australia. Seeds were sown in seedling germination trays. Forty-five days old seedlings were transferred to field at the Department of Plant Breeding and Genetics, PMAS-Arid Agriculture University, Rawalpindi (33.5651°N and 73.0169°E). The experiment was laid out in Randomized Complete Block Design (RCBD) with three replicates. After transplantation all standard field experimental protocols and cultural practices were performed. The row to row (R×R) distance was maintained at 15–20 cm and plant to plant (P×P) distance was 10–15 cm. Twelve plants (bulbs) per replicate per genotype were harvested randomly and stored under dark condition for further analysis.

### FTIR analysis

Bulbs of stored onions were randomly selected from three technical replicates. Eight bulbs per genotype/replicate were used for analysis. Each onion bulb was slightly peeled to remove dry skin, cut into quarters. Ten gram of each sample was oven dried (65°C) for 24 h and ground into a homogenized powder by using pestle and mortar. These samples were stored in Eppendorf`tubes at 4°C for FTIR (Fourier Transform Infrared Spectroscopy) analysis. One hundred microgram of each powdered sample was placed into the sample holder and spectra were recorded in the range of 4000–400 cm^-1^ in an FTIR spectrophotometer (Tensor 27, Bruker, equipped with ZnSe ATR). The bands were identified by observing vibration of sample atoms when these were exposed to infrared region of electromagnetic spectrum and showed in wave number (cm^-1^).

### Development of crosses

Five high yielding genotypes (Super Sarhad, MKS-77127, MKS-132807, MKS-TPSWP and MKS-14278) used as lines (female parent). The seven genotypes with high concentration of nutritional and pharmaceutical content (MKS-636ZU, MKS-8823GO, Onion Swat, Phulkara, 28540, 28539 and MKS777) were used as testers (male parent). Lines and testers were planted in a crossing block (season 2017) following line × tester crossing fashion to develop 35 crosses for subsequent analysis/evaluation.

The emasculation was done manually by removing the anthers of three fully mature umbels of each line by selecting eighty florets within each umbel. The emasculated umbels were covered with butter paper bags. The pollination was done manually by dusting the pollen grains with the help of camel hairbrush. The seeds of F_1_ crosses were harvested, cleaned and stored.

The harvested seeds of F_1_ crosses along with their parents were sown during 3^rd^ week of Oct 2017 in germination trays filled with potting media containing soil, compost, and sand (1:1:1). The seedlings were transplanted in field after 45 days of germination by keeping P×P and R×R distance 10 cm and 15 cm, respectively. Before transplanting nursery in field, soil properties of field were studied and analysis revealed that Nitrogen (N), Phosphorus (P) and Potassium (K) were present in range of 87.8 (ppm), 78.44 (ppm) and 71.79 (ppm), whereas PH and EC were recorded 7.74 and 0.0321 (ds/m). The genotypes were assigned in field by using Randomized Complete Block Design (RCBD) containing 35 crosses and their 12 parents. The data were collected and subjected to the analysis of heterosis (Both mid and better parent), general and specific combining ability (GCA and SCA) and variance components for nutraceutical components. The data were analyzed using R Program (version 3.2.1).

### Determination of nutraceutical components

The pyruvic acid contents were determined according to Anthon and Barrett by using dinitro phenyl hydrazine (DNPH) reagent [17]. Vitamin C content were determined by following the method of AOAC (Association of Official Analytical Chemists) [18]. Total phenolic contents (TPC) were determined using the Folin-Ciocalteu reagent, according to Zhang Shi-lin et al [19] and Total flavonoid content (TFC) were estimated by using 1g of each ground spice from oven dried onion samples followed by Kaur and Kapoor [20].

### Statistical analysis

Data obtained from FTIR spectra were used to obtain mean and standard deviation (SD). The data of nutraceutical components were subjected to analysis of variance (ANOVA) followed by least significant difference (LSD) using R-project (version 3.4.1).

## Results

### Screening of onion genotypes for nutraceutical components through FTIR

The FTIR spectra of onion genotypes were shown in the S1 Fig. The data on peak values and functional groups as analyzed by FTIR showed strong characteristic absorption bands at different wavelengths (Table 1). Genotypes showed considerable differences for these characteristic bands due to the variation among functional groups that assigned to these bands. The structural formulas of chemicals provided information about functional groups that were helpful for identification of specific location of chemicals over different wavelengths and absorbance, while comparing with infrared (IR) spectrum. When functional groups were compared with IR chart (infared spectrum chart), they provided range and intensity of different chemicals.

**Table 1:** Accessions showing the presence of different functional groups at different wave lengths indicating different phytochemicals.

No.	Genotypes	Onion Bulb Color	Functional Groups							
			Nitrile(C = N Stretch)	Alcohol(OH Stretch)	Amine(N-OH Stretch)	Carboxylic Acid(O-H Stretch)	Aromatic(C = C Bending)	Amide(N-OH Stretch)	Alkenyl(C = C Stretch)	Ketone(C = O Stretch)
1	32813	Light Brown	-	+	+		+	-	+	+
2	MKS0502	Yellowish Brown	-	+	+	+	+	+	-	-
3	MKS077127	Brown	-	+	+	+	+	+	-	-
4	MKS132807	Brown	-	-	+	+	+	+	-	+
5	MKS0103GB	Dark Brown	-	+	+	+	-	+	+	-
6	28533	Light Brown	-	+	-	+	+	+	-	-
7	28535	Dark Brown	-	-	+	+	+	-	-	-
8	MKS050103GW	White	-	+	+	+	+	-	-	-
9	28529	Brown	-	+	+	-	+	+	-	+
10	Super Sarhad	Light Brown	+	+	+	+	+	-	-	-
11	28534	Brown	-	+	+	+	+	+	-	-
12	MKS0TPSWP	Yellowish Brown	-	-	+	+	+	+	+	+
13	MKS777	Purplish Brown	-	+	+	+	-	-	+	+
14	MKS636ZU	Purplish Brown	-	+	+	+	-	-	+	+
15	28538	White	-	+	+	+	-	+	+	+
16	MKS014278	Dark Brown	-	-	+	+	-	-	+	+
17	28537	Purplish Brown	-	+	+	+	+	+	+	-
18	Phulkara	Yellowish Brown	-	+	+	+	-	-	+	
19	CGN18750	White	-	+	-	+	-	+	+	+
20	28530	Light Brown	-	+	+	+	+	+	-	-
21	MKS5021	Purplish Brown	-	+	+	+	-	-	+	+
22	Sand	Light Brown	+	-	+	-	+	-	-	+
23	CGN16350	White	-	+	-	+	-	+	+	+
24	CGN20182	Brown	-	+	-	+	-	-	-	+
25	MKS8823GO	Purplish Brown	-	+	+	+	-	+	+	+
26	28531	Light Brown	+	-	+	-	+	-	-	+
27	MKS1290SGB	Yellowish Brown	-	+	+	-	-	+	+	-
28	28532	Light Brown	-	+	+	-	+	+	+	-
29	28539	Light Brown	-	+	+	+	+	+	+	-
30	170	White	-	+	-	+	-	+	+	+
31	NARC-2005	Purplish Brown	-	-	+	+	-	+	+	-
32	CGN15740	Light Brown	-	-	-	+	+	+	+	-
33	CGN24762	Light Brown	+	+	-	+	+	+	-	-
35	28540	Light Brown	-	+	+	-	+	+	+	-
36	171	White	-	+	-	+	-	+	+	+
37	Onion Swat	Purplish Brown	-	+	-	+	-	+	-	-
38	28536	Light Brown	-	+	+	-	+	+	+	-
39	MKSRDFE	Light Brown	-	+	-	+	-	-	+	-

Wave numbers are from Tensor equipment with a scan range from 400 to 4000 cm^-1^ with a resolution of 4 cm^-1^ range.

### Grouping of onion genotypes based on common bulb color and functional groups

Genotypes were categorized into six groups based on common bulb colors and functional groups (S2 Fig). Genotypes in group I (White color) had common functional groups alcohol/phenol OH stretch, carboxylic acid O-H and aromatic bending C = C (Table 2) suggesting that these functional groups were the major sources of proteins, anthocyanin, vitamin C, carbohydrates, phenols and polyphenols [21].

**Table 2:** Classification of genotypes based on common colors and functional groups.

Genotype	Color	Common Functional Groups	Color Group
MKS050103GW	White	Alcohol/phenol O-H stretch;	Group I
28538		Carboxylic Acid (O-H);	
CGN16350		Aromatic C = C Bending	
170			
171			
CGN-18750			
MKS0502			
MKS1290SGB	Yellowish Brown	Alkenyl/Phenol O-H stretch;	Group II
MKS0TPSWP		Amine (N-H stretch)	
Phulkara			
MKS132807			
MKSRDFE	Light Brown	Alcohol/Phenol OH Stretch;	Group III
Super Sarhad		Amine N-H Stretch;	
28540		Carboxylic Acid (OH) group;	
28536		Amide (N-H Stretch);	
CGN15740		Ester;	
CGN24762		Aromatic C = C Bending	
28533			
28532			
28531			
28530			
28539			
Sand			
MKS014278	Dark Brown	Phenol/Alcohol O-H Stretch;	Group IV
		Amine N-H Stretch;	
MKS0103GB		Amide N-H Stretch;	
		Aromatic C = C Bending;	
28535		Carboxylic Acid	
MKS5021	Purplish Brown	Phenol OH Stretch	Group V
MKS777		Amide N-H Stretch	
Onion Swat		Amine N-H Stretch;	
NARC-2005		Carboxylic Acid	
28537			
28529			
28534	Brown	Alcohol/Phenol OH Stretch,	Group VI
CGN20182		Amine N-H Stretch,	
28529		Carboxylic Acid OH Stretch,	
MKS077127		Alkenyl	
MKS132807			

The group II (yellowish brown) had common functional groups phenol O-H Stretch, and amine N-H Stretch indicating the bands at different wave lengths due the presence of O-H group, N-H stretch, C = 0 and C = C bending. The presence of these functional groups suggested that these genotypes were abundant in alcohol or phenol carbohydrates, lipids and polyphenols (Table 2).

The common members of Group IV (Dark brown) had alcohol/phenol OH stretch, amide N-H stretch, aromatic C = C Bending and carboxylic Acid. The group V (Purplish brown) and group VI (Brown) comprised of most common groups alcohol/ phenol O-H stretch, carboxylic acid and amide N-H stretch. The most common functional groups of group VI were alcohol/phenol O-H stretch, amine N-H stretch, carboxylic acid OH, stretch, ester and aromatic C = C bending (Table 2).

### Quantification of nutraceutical components

#### Pyruvic acid (μM)

The pyruvic acid content ranged between 0.2μM- 17.18 μM and highly significant variation was recorded. The highest content was recorded for MKS777, Onion Swat and lowest for 28531 and 28540 (Tables 3 and 4).

**Table 3:** Analysis of variance showing significant differences among 39 onion genotypes for nutraceutical traits.

SOV	DF	Pyruvic Acid Content	Vitamin C	Total Flavonoid Content	Total Phenolic Content
Blocks	2	17.59	10.18	84.5	2.42
Genotypes	38	392.29**	10.18**	84.50**	2334.12**
Error	76	4.02	0.7	2.09	0.01
CV		2.96	6.06	3.43	3.03

**Significant level<0.01

**Table 4:** Mean performance of 39 onion genotypes for four nutraceutical traits.

Genotypes	Pyruvic Acid Content	Vitamin C	Total Flavonoid Content	Total Phenolic Content
32813	8.26	0.45	1.08	0.173
MKS0502	4.74	0.11	0.36	0.81
MKS077127	8.91	0.42	2.12	0.70
MKS132807	8.49	0.16	1.97	1.34
MKS0103GB	7.33	0.34	0.45	0.4
28533	13.82	0.43	2.3	0.19
28535	10.49	0.19	0.43	0.17
MKS050103GW	6.6	0.5	1.93	1.44
28529	7.54	0.28	1.146	0.12
Super Sarhad	9.32	0.34	1.76	0.34
28534	9.02	0.38	0.42	0.186
MKS0TPSWP	9.06	0.48	1.64	2.02
MKS777	17.10	0.45	1.53	0.16
MKS636ZU	8.193	0.5	2.17	0.66
28538	7.60	0.28	1.18	0.23
MKS014278	7.00	0.49	2.02	0.67
28537	7.43	0.33	1.17	0.29
Phulkara	16.99	2	2	1.45
CGN18750	7.57	0.17	0.72	0.07
28530	7.64	0.27	1.30	0.34
MKS5021	7.44	0.45	1.13	0.38
Sand	7.65	0.34	0.6	0.45
CGN16350	7.56	0.49	0.71	0.39
CGN20182	7.31	0.4	0.63	0.37
MKS8823GO	16	2.5	2.56	1.39
28531	0.42	2.4	1.75	0.69
MKS1290SGB	7.75	0.42	1.49	0.04
28532	7.68	0.11	0.36	0.8
28539	17	2.23	2	1.46
170	7.36	0.28	1.4	0.46
NARC-2005	7.49	0.49	1.5	0.48
CGN15740	7.43	0.33	1.26	0.45
CGN24762	7.69	0.27	0.84	0.44
28540	0.23	2.26	2.0	0.7
171	7.55	0.33	0.31	0.06
Onion Swat	16.7	2.01	2.01	1.59
28536	7.77	0.29	0.159	0.45
MKSRDFE	7.75	0.26	1.15	0.26

#### Vitamin C (mg/100ml)

The vitamin C content showed highly significant variation among genotypes. The vitamin C content range between 0.11–2.5mg/100 ml. The highest vitamin C content were recorded for MKS8823GO and lowest for 28532 (Tables 3 and 4).

#### Total flavonoid (QE/g)

The total flavonoid contents were measured during present study and highly significant variation was observed among genotypes. These contents range from 0.15–2.56 (QE /g). The maximum value was recorded for MKS8823GO, and minimum was observed in 28536 (Tables 3 and 4).

#### Total phenolic (GAE/g)

The highly significant variation was observed for total phenolic contents during current study. Phenolic content range between 0.04–2.02 GAE/g. The highest phenolic content was recorded for MKS-TPSWP and lowest were recorded for 171(Tables 3 and 4).

### Combining ability, variances, heterosis and heritability

#### Pyruvic acid content

All lines and testers showed non-significant general combining ability (GCA) values, while specific combining ability (SCA) values were expressed significantly for all combinations except of MKS132807 × MKS636 ZU (-0.258) (Table 5). The highest SCA value was obtained for MKS-14278 x MKS777 (0.505) while the lowest value was found for Super Sarhad x MKS777 (0.115).

**Table 5:** Estimates of general combining ability (GCA) and specific combining ability (SCA) effects of parents for 4 different traits based on Line× Tester in onion.

Combining Ability	Pyruvic acid content	Vitamin C content	Total Flavonoid Content	Total Phenolic content
GCA For Lines				
MKS-14278	8.9E-16^NS^	-2.2E-02^NS^	-5.8E-02^NS^	-0.02232^NS^
MKS-77127	-1.2E-15^NS^	-3.0E-02^NS^	-6.7E-02^NS^	-0.03023^NS^
MKS-TPSWP	-1.7E-15^NS^	2.7E-02^NS^	9.8E-02^NS^	0.026721^NS^
MKS132807	9.1E-16^NS^	4.4E-02^NS^	-4.1E-03^NS^	0.044111^NS^
Super Sarhad	1.0E-15^NS^	-1.8E-02^NS^	3.1E-02^NS^	-0.01828^NS^
**GCA For Testers**				
28539	-1.8E-14^NS^	0.0E+00	5.4E-02^NS^	0
28540	1.7E-14^NS^	0.0E+00	2.7E-02^NS^	0
MKS636ZU	-2.5E-14^NS^	0.0E+00	1.6E-02^NS^	0
MKS777	8.7E-14^NS^	0.0E+00	-2.2E-02^NS^	0
MKS8823GO	-3.1E-14^NS^	0.0E+00	-3.9E-02^NS^	0
Onion Swat	1.8E-14^NS^	0.0E+00	-1.9E-02^NS^	0
Phulkara	-4.7E-14^NS^	0.0E+00	-1.6E-02^NS^	0
**SCA For Crosses**				
Super Sarhad x MKS636 ZU	0.098**	-0.022^NS^	-0.340^NS^	-0.06022^NS^
Super Sarhad x MKS8823GO	-0.146^**^	0.476^NS^	0.439^NS^	0.641643**
Super Sarhad x onion swat	0.108**	-0.316^NS^	-0.293^NS^	0.275452^NS^
Super Sarhad x Phulkara	0.505**	-0.164**	-0.142^NS^	-0.0704**
Super Sarhad x 28540	0.108**	-0.316^NS^	-0.239^NS^	-0.26366**
Super Sarhad x MKS777	-0.115**	-0.246^NS^	-0.053^NS^	-0.25756**
MKS-77127 x MKS636 ZU	0.057**	-0.309^NS^	-0.098**	0.041496^NS^
MKS-77127 x MKS8823GO	-0.197**	-0.279**	-0.123^NS^	0.075063**
MKS-77127 x onion swat	-0.329**	0.281^NS^	0.215^NS^	-0.2128**
MKS-77127 x Phulkara	0.332**	-0.309^NS^	-0.236^NS^	-0.35226**
MKS-77127 x 28540	-0.228^**^	-0.197^NS^	-0.107^NS^	-0.27862^NS^
MKS-77127 x MKS777	-0.024**	0.332^NS^	-0.064**	-0.30914^NS^
MKS-77127 x 28539	-0.126**	-0.014^NS^	-0.079^NS^	-0.30914^NS^
MKS-TPSWP x MKS636 ZU	-0.289**	0.336**	0.307*	-0.14229*
MKS-TPSWP x (MKS8823GO)	-0.167**	-0.346**	0.333^NS^	0.203549**
MKS-TPSWP x onion swat	-0.268**	-0.142^NS^	-0.174^NS^	0.010284^NS^
MKS-TPSWP x Phulkara	-0.289^NS^	0.336^NS^	0.383^NS^	0.0408^NS^
MKS-TPSWP x 28540	-0.177**	0.204^NS^	0.146**	-0.34573^NS^
MKS-TPSWP x MKS777	0.098**	0.010^NS^	-0.159**	0.335783^NS^
MKS-TPSWP x 28539	0.332**	0.041^NS^	-0.112**	0.335783^NS^
MKS132807 x MKS636 ZU	-0.258^NS^	0.003**	0.511^NS^	0.145477**
MKS132807 x MKS8823GO	0.230**	-0.150^NS^	-0.255**	0.226851^NS^
MKS132807 x onion swat	0.006**	0.145^NS^	0.447^NS^	0.033586^NS^
MKS132807 x Phulkara	0.006**	0.145^NS^	-0.263**	0.318398^NS^
MKS132807 x MKS777	0.230^NS^	0.227*	-0.291**	0.145477**
MKS132807 x 28540	0.484**	0.034**	0.116^NS^	-0.14951**
MKS132807 x 28539	-0.289**	0.318^NS^	-0.296^NS^	0.003071^NS^
MKS-14278 x MKS636 ZU	0.179^**^	-0.318^NS^	0.456**	-0.31603^NS^
MKS-14278 x (MKS8823GO)	0.484**	0.096^NS^	0.034*	-0.31603^NS^
MKS-14278 x onion swat	0.179**	-0.319^NS^	0.046^NS^	-0.24584^NS^
MKS-14278 x Phulkara	0.484**	0.096**	-0.089*	0.22206**
MKS-14278 x 28540	-0.309**	0.238^NS^	-0.123^NS^	0.476356^NS^
MKS-14278 x MKS777	-0.228**	0.025**	-0.142^NS^	-0.16447**

The variance components including dominant, additive, genotypic and environmental variance were calculated by using line × tester mating design (Table 6). The dominant and additive variances showed similar values i.e. 0. 279, respectively. The genotypic and environmental variances were 0.070 and 2.06×10^−5^, respectively. The line, tester, and line ×tester variance was 1.56×10^−16^, 5.81×10^−15^ and 0.069, respectively.

**Table 6:** Estimates of broad sense, narrow sense heritability and variance components for four nutraceutical traits.

Trait	Dominant variance	Additive variance	Genotypic Variance	Environmental Variance	Line Variance	Tester Variance	Line×testers Interaction	Gene action	Broad sense heritability	Narrow sense heritability
Pyruvic acid content	0.27	0.27	0.06	2.06×10^−5^	1.56×10^−16^	5.81×10^−15^	0.06	Dominant-Additive	0.99	0.49
Vitamin C	0.24	0.25	0.06	1.88×10^−5^	0.003683	0.1	0.06	Additive	0.99	0.51
Total Flavonoid content	0.27	0.32	0.08	2.42×10^−5^	0.009488	0.004488	0.06	Additive	0.99	0.53
Total Phenolic content	0.311	0.31	0.07	2.3×10^−5^	2.61×10^−16^	3.43×10^−18^	0.07	Dominant-Additive	0.99	0.49

Heterosis and heterobeltiosis were calculated for pyruvic acid content. The heterobeltiosis ranged from 17.603% to -2.912% for 35 crosses, while heterosis was found 26.1469% to 13.82% for all the 35 crosses. Non-significant differences were observed for parents vs crosses and lines ×Testers, while the significant differences were observed for lines and testers (Table 7). The value of broad sense heritability (0.99%) was higher than that narrow sense heritability (0.49%) for pyruvic acid content (Table 6).

**Table 7:** Analysis of variance for parent’s vs crosses indicating significance level of heterosis for nutraceutical traits.

Source of Variation	Df	Pyruvic Acid Content	Total Flavonoid Content	Vitamin C	Total Phenolic content
**Replications**	2	3.67E-02*	0.117755*	0.00101627^NS^	55.30289*
**Treatments**	46	5.33E-02*	4.015052**	2.067244**	388.6539**
**Parents**	11	2.10E-01**	1.446537**	0.0487233**	528.9077**
**Parents vs crosses**	1	5.81^NS^	109.0098**	29.175938**	475.1988**
**Crosses**	34	4.085266e-03^NS^	1.757959**	1.922298**	340.7322**
**Lines**	4	6.686667e-03**	3.197933^NS^	13.157911**	1489.795**
**Testers**	6	1.83E-02**	4.909912*	1.08394762*	865.97148^NS^
**Lines×Testers**	24	9.333333e-05^NS^	0.7299**	0.260251**	17.911*
**Error**	92	5.62E-02	0.103903	0.004384	52.43008
**Total**	140				

**Significance level<0.01

*Significance level<0.05

#### Vitamin C

The non-significant differences were observed for GCA (lines and testers), while highly significant differences were recorded among crosses for SCA except of few. The highest SCA was observed for MKS132807 x MKS777, (0.227), while lowest was expressed in MKS132807 x MKS636 ZU (0.003) (Table 5). The dominant and additive variances were calculated as 0.24 and 0.25, respectively. The genotypic and environmental variances were 0.06 and 1.88×10^−5^, respectively. Line, tester, and line ×tester variances were 0.003, 0 and 0.06, respectively (Table 6).

The average heterobeltiosis for vitamin C content ranged from 37%-99.9%, while the significant positive heterobeltiosis was recorded for MKS-14278 x 28540(98.13%), Super Sarhad ×28540 (94.25%) and for Super Sarhad×28540(93.57%) etc. The average mid parent heterosis was recorded from 83.34%-99.99% for all the 35 crosses. The ANOVA showed highly significant differences for vitamin C content (Table 7). Maximum significant mid parent heterosis was recorded for MKS-14278×Phulkara (99.99%), Super Sarhad×28539(99.99%) and for Super Sarhad×Onion Swat (99.98%) (Table 8). The broad sense heritability (0.99%) was found greater than narrow sense heritability (0.51%) (Table 6).

**Table 8:** Estimation of mid and better parent heterosis for four nutraceutical components.

Crosses	Pyruvic acid content		Vitamin C		TF content		TP content	
	MPH	BPH	MPH	BPH	MPH	BPH	MPH	BPH
Super Sarhad x MKS636 ZU	16.14**	15.53*	96.73*	93.57*	95.54*	33.66*	4.48**	-8.41*
Super Sarhad x (MKS8823GO)	16.72**	13.43*	99.98*	74.98*	99.26*	39.44*	4.18**	-6.81*
Super Sarhad x onion swat	17.03**	6.76**	99.99*	231.4*	99.23*	61.01*	5.90**	-13.16*
Super Sarhad x Phulkara	18.97**	0.09*	99.98*	79.91*	99.89*	89.52*	5.05**	-9.69*
Super Sarhad x 28540	22.58**	-19.93*	100.0*	94.26*	99.63*	97.22*	4.46*	-2.81*
Super Sarhad x MKS777	18.4**	3.42**	99.9**	80.30*	98.87*	34.05*	4.05**	-4.48*
Super Sarhad x 28539	17.13**	3.42**	100.0*	91.62*	99.60*	63.32*	4.59***	1.67***
MKS-77127 x MKS636 ZU	13.82**	9.22**	229.8*	192.2*	98.83*	43.17*	4.83*	-26.73*
MKS-77127 x (MKS8823GO)	14.30	6.19	211.54	202.29	99.83	47.79	4.47	-25.99
MKS-77127 x onion swat	14.44**	0.12**	180.8*	116.5*	99.72*	73.65**	7.04**	-2.31***
MKS-77127 x Phulkara	16.06**	-5.95*	240.5*	178*	99.47*	92.55*	5.79*	-8.27*
MKS-77127 x 28540	23.39**	-21.12*	224.7*	199.7*	99.21**	84.74**	4.84**	-23.74*
MKS-77127 x MKS777	15.67	-2.91	240.29	179.10	99.42	41.32	6.93	-12.76
MKS-77127 x 28539	14.45**	-2.91*	223.0*	202.1*	99.08*	75.50*	5.04**	-21.29*
MKS-TPSWP x MKS636 ZU	14.63**	8.93**	661.9**	659.7*	99.50**	31.47**	3.12**	-4.48*
MKS-TPSWP x (MKS8823GO)	18.78**	16.68**	537.63**	448*	99.29*	34.14*	4.26**	-1.32*
MKS-TPSWP x onion swat	15.40**	9.96**	311.1**	191.0**	99.26**	52.82**	5.88**	-17.11*
MKS-TPSWP x Phulkara	17.30**	2.63***	391.71**	350.08**	99.86*	77.51**	5.09*	-14.04*
MKS-TPSWP x 28540	26.15**	-15.69*	337.57**	317.07*	99.63**	84.19*	4.52*	3.17*
MKS-TPSWP x MKS777	16.81**	6.30**	746.14*	676.20*	99.97*	30.13*	4.15*	-9.44*
MKS-TPSWP x 28539	19.43**	9.96**	428.69*	397.39*	99.60*	54.82*	4.65*	1.30**
MKS132807 x MKS636 ZU	17.01**	2.32**	84.77*	79.99*	98.99*	46.86*	3.59*	0.75*
MKS132807 x MKS8823GO	17.70**	6.07*	87.13**	65.19***	98.73**	50.85*	3.30*	2.79**
MKS132807 x onion swat	18.13*	13.30*	90.91*	37.56**	99.89*	79.82*	4.50*	-21.10*
MKS132807 x Phulkara	20.50*	14.58*	83.35*	63.46**	99.60*	85.67*	3.81*	-18.33*
MKS132807 x MKS777	19.84*	18.99*	85.54*	81.96*	99.33*	78.42*	3.51*	-0.34*
MKS132807 x 28540	25.49*	-11.86*	83.39**	63.84**	99.61*	44.86*	4.66*	-12.79*
MKS132807 x 28539	18.29	17.60	85.67	79.65	99.22	81.82	3.58	-4.49
MKS-14278 x MKS636 ZU	15.56*	12.23*	100.00*	89.39*	99.13*	39.07*	4.05**	-1.87*
MKS-14278 x MKS8823GO	16.13**	15.59*	100.00*	81.21*	98.89*	42.40*	3.77**	0.05**
MKS-14278 x onion swat	16.40*	8.58*	100.00*	46.79*	99.92*	66.55*	5.18*	-18.80*
MKS-14278 x Phulkara	18.34	1.58	100.00	73.81	99.65	96.53	4.44	-15.84
MKS-14278 x 28540	21.91**	-19.44*	100.09**	98.14**	99.39**	94.21*	4.01*	3.35*
MKS-14278 x MKS777	17.83*	5.08*	300.00*	248.34*	99.68*	37.40*	3.56*	-11.41*
MKS-14278 x 28539	16.48**	5.08*	100.01*	100.81*	99.31*	68.21*	4.11*	-1.08*

#### Total flavonoid content

The non-significant GCA differences were shown by line×tester for total flavonoid content, while highly significant differences were determined for SCA among maximum number of crosses. The high SCA value was recorded for MKS-1478×MKS636ZU (0.456) and low value was observed for MKS-77127 x MKS636 ZU (-0.09) (Table 5). The additive and dominant variances were calculated as 0.32 and 0.27, respectively. The genotypic and environmental variances were 0.08 and 2.42×10^−5^, respectively. The variances for line, line×tester and line×tester interactions were 0.09, 0.004 and 0.06, respectively (Table 6).

Average heterobeltiosis for total flavonoids was recorded as 97.21% to 30.13% for 35 crosses (Table 8). The maximum significant positive heterosis was recorded for three crosses for Super Sarhad × 28540 (97.21%), MKS-14278 × Phulkara (96.52%) and for MKS-14278 × 28540 (94.21%). Significant differences for total flavonoids were observed among the genotypes (Table 7). The significant differences were also observed for parents, parents vs crosses for lines and also for line×testers while non-significant differences were observed for testers.

#### Total phenolic content

The GCA effects were found non-significant for line×tester, while highly significant SCA differences were measured for maximum number of crosses. The high SCA value was recorded for Super Sarhad×MKS8823GO (0.641) and low was observed for MKS132807×28540(-0.14) (Table 5). The dominant and additive variances for total phenolic content were calculated similar as 0.3. The genotypic and environmental variances were 0.07 and 2.3×10^−5^, respectively. The line, tester and line×tester interactions were 2.61×10^−16^_,_ 3.43×10^−18^ and 0.07, respectively (Table 6).

The ANOVA shows highly significant differences for total phenolic content (Table 7). The average heterobeltiosis for total phenolic content ranged from 3.35–26.72%, while the significant positive heterobeltiosis was recorded for MKS-14278 × 28540 (3.35%), MKS-TPSWP ×28540 (3.174%) and for MKS132807 × MKS8823GO (2.78%). Average mid parent heterosis was recorded for 7.04% -3.12% for all the 35 crosses (Table 8). The maximum positive significant mid parent heterosis was recorded for MKS-77127 × Onion Swat (7.04%), MKS-77127 × MKS777 (6.92%) and for Super Sarhad × Onion Swat (5.90%), while the maximum positive significant heterobeltiosis was recorded for MKS-14278 × 28540 (3.35%), MKS-TPSWP × 28540 (3.17%) and for MKS132807 × MKS8823GO (2.78%). The broad sense heritability (0.99%) was found greater than narrow sense heritability (0.53%) (Table 6).

## Discussion

Onion has been used as a condiment as well as medicine since ancient times. The current study was planned to screen out the existing germplasm collection for nutraceutical traits and to understand the genetics of various nutraceutical components. Germplasm was screened for nutraceutical contents (pyruvic acid, vitamin C, Total flavonoids and Total phenolics) using FTIR and spectrophotometery/colorimetry. The screened genotypes were then crossed following line × tester technique. The obtained crosses /combinations were also tested for their nutraceutical contents (Tables 3–8)

### Nutraceutical profiling through FTIR

Pyruvic acid acts as an indicator for determination of pungency. Genotypes were divided into six different groups based on common bulb color and functional groups (Described in Result Section) and we found a significant association among bulb colors and pyruvic acid content as light-colored genotypes were found with lower pungency and vice versa as revealed by FTIR as well as spectrophotometry. FTIR spectrum (Range 4000–515 cm^-1^ S1 Fig) have shown scan range of detected chemicals. The key feature of the spectrum are absorbance or vibrational modes associated with pyruvic content in wave range of 1700–1000 cm^-1^, however, variation exists among genotypes for wavenumbers and vibrational modes indicating presence of pyruvic acid. Previous study reported that there are no distinct spectral features of onion that are consistently associated with pyruvate, they observed peak of carboxylate functional group at 1700 cm^-1^_,_ while this peak is absent or merged with other constituents at 1600 cm^-1^ indicating low concentration of pyruvate [22]. During current study we have found that vitamin C content fall within fingerprint region of 1200-1600cm^-1^ in spectrum of onion genotypes with different vibrational modes (1003.65,1041.49,1184.92 etc.) indicating intensity of vitamin C content. Nevertheless, vibrational modes may vary among genotypes. FTIR spectrum revealed presence of TP content in fingerprint region of 3000-3500cm^-1^ with different vibration modes predicting intensity of TP content (high, low or moderate). Previous study has reported use of FTIR analysis for detection of bioactive compounds in fresh onion leaves and similar to current observation they observed that methanolic extract is more active for detection of bioactive compounds including phenols and vitamin C and contrary to our finding they observed these compounds in the infra-red radiation range of 600–4000 cm^-1^ [23].

We have recorded flavonoid content in fingerprint region of 2500–2999 cm^-1^ with different vibrational modes or peaks that may vary among genotypes. Previously, quercetin content was recorded in spectrum of onion genotypes of three different colors (red, yellow, and white). The frequency range was 400–4000 cm^-1^, however, there was no clear difference in spectra of three genotypes [24].

### Quantification of nutraceutical components

This study casts an insight over the importance of pyruvic acid content. The pungency is an important characteristic of onion. Mostly, consumers do not prefer the onion with high pungency, however, this preference varies among populations/countries. People in western countries prefer less pungent while south Asian people like onion with high pungency [25]. Thus, the measurement of pyruvic acid content provides a relative degree of pungency in onion bulb. The onion genotypes used in this study were grouped in various colors viz. White, Yellowish Brown, Light Brown, Brown, Dark Brown, and Purplish Brown. Our observations establish a relationship between bulb color and pungency/pyruvic acid contents as lighter color was found associated with lower pungency and vice versa as revealed by FTIR as well as spectrophotometry (Tables 2 and 3). These observations are in concurrence with previous findings [26]. A significant variation in PA suggests the involvement of genetic effect as evident from inheritance studies where genotypic variance was quite higher than environmental variance. This indicates the lesser influence of environment over PA content. Our findings are supported by the observations by Yoo et al [27]. They observed small impact of environment including soil sulfur content, indicating it as a strongly inherited trait. Further, non-additive type of gene action was predicted as SCA was significant [28, 29].

Vitamin C acts as a free radical scavenger with the ability to scavenge free radicals and other oxygen reactive species (ROS). During current study, vitamin C content were measured in 39 onion genotypes of six different colors. Results indicated that white onion had lowest vitamin C content as compared to colored onions, however, a mixed behavior was observed among colored ones. Some genotypes of purplish brown group had high concentration of vitamin C, while rest of them had low concentration. Similar trends were observed for light brown and yellowish-brown genotypes (Tables 2 and 3). These results are in concurrence with the observations of Mlcek *et al* [30] who observed a weak association of vitamin C content with bulb color. This suggests that improving vitamin C content in onion bulb could be done by recurrent selection by focusing on suitable combination of color and vitamin C content. The greater genetic variance and high heritability further supports the proposed strategy as environmental factors would have less impact over vitamin C Chattopadhyay *et al*. [31].

Flavonoids are very important from medicinal point of view due to their anti-hypertensive and anti-cholinesterase activity [32]. Currently, onion genotypes of different colors were characterized for total flavonoid (TF) content and variation was observed based on colors. The Purplish brown genotypes were found with highest concentration of total flavonoids, while intermediate concentration was observed in yellowish brown genotypes. The least concentration of total flavonoids was estimated in white colored genotypes. Our current findings agree with previous study which determines highest flavonoids concentration in red skin color as compared to yellow skinned genotypes. Further, they observed there is negligible quantity of TF in white genotypes [33, 34]. Our study indicated that variation among genotypes for TF content is due to genetic factors because greater genetic variance we observed as compared to environmental variance. However, several studies conducted to understand the impact of environment over flavonoid content demonstrated that despite of being genetically controlled trait, environment influences the quality and quantity of TF in onion [34, 35]. Further, non-additive type of gene action was observed for TF content in current study. Thus, results of current and previous observations suggested that appropriate selection of parents (preferably dark colored), having good specific combination, could be helpful for improvement of TF content. A delayed selection would be more fruitful.

Total phenolic content (TP) is considered as medicinally important and its high concentration gives high nutraceutical value to onions. We observed TP content in a collection of onion genotypes and found that highest TP concentration purplish brown and yellowish brown genotypes. The genotypes of rest of the colored groups had low TP content, while white onion had lowest TP content. Previously, Gökçe *et al*., [36] also reported highest TP content in yellow onions. Moreover, it is obvious from previous findings that phenolic contents are responsible of brownish shade in fruits. Moreover, variation in TP content was due to genetic potential as it is indicated higher genotypic variance as compared to environmental variance. Contrary to our results MPO fu et al., [37] observed significant impact of both genetic and environmental variance over TP content in hard spring wheat. Further, they suggested that genetic variance for TP content indicated that selection would be possible for these in quantitative breeding programs but significant environmental variance may be delayed or complicated this process. Anntonen et al. [38] also found significant effect of genotypic and environmental variance over TP content in Raspberry plant. Our study observed that TP content are controlled by dominant type of gene action as SCA effect is significant. Kaushik *et al*., [39] estimated additive type of gene action for TP content in eggplant. It is obvious from previous and current observation that TP content could be improved through an efficient selection.

### Estimation of heterosis for nutraceutical components

The estimation of combining ability effect is of crucial importance to achieve desired crosses with high magnitude of heterosis. General and specific combining ability estimates were executed for nutraceutical components during current study. We have analyzed non-significant GCA effect for lines as well as for testers for all crosses indicating the significance of non-fixable type of gene action in the expression of all these traits. While this study reported significant SCA effect for all the crosses except of few denoting non-significant SCA effect showing that these combinations can be successfully utilized for obtaining frequency of desirable alleles. Patil and one of his colleagues [40] examined combining ability effect on yield and quality parameters including pyruvic acid content of white onion and their results were contrary to our current findings. Previously Abhishek et al., [41] reported combining ability effect for flavonoids and phenolics in cabbage and similar to our study they calculated significant SCA effect, but they also recorded significant GCA effect that is not in agreement with our observations.

Heterosis is thought to be a crucial factor to increase variability, heritability, and genetic diversity for various nutraceutical components. It enhances heterozygosity in a hybrid due to superior gene content contributed by both parents in a hybrid. The exploitation of heterosis introduced several ways to obtain good quality traits. The superior hybrids can be identified through nature and magnitude of heterosis. Currently, mid and heterobeltiosis (better parent) were measured for studied traits and heterobeltiosis was estimated lower than mid parent heterosis for pyruvic acid content. The reason for low heterobeltiosis was combination of average and good parent or may be due to poor and average parents. However, this indicates that combinations with maximum mid parent heterosis could be exploited for further improvement of pyruvic acid content. Rafiq *et al*., [42] observed mid parent heterosis percentage for pyruvic acid content that is contrary to our findings. Hybrids with mid and better parent heterosis were found with increased concentration of total flavonoid content. However, heterobeltiosis was found lower than mid parent heterosis this could be associated with combination of poor or average parent with best one. The selection of hybrids with maximum mid parent heterosis could be fruitful for future enhancement of total flavonoid content.

Genetic basis of flavonoids was not studied previously, however heterotic value for bioactive compounds including total flavonoids was found lower in onion by Faria *et al*., [43]. We observed positively significant average heterosis percentage and negatively significant heterobeltiosis for maximum number of cross combinations for total phenolic content. The negative heterobeltiosis may be due to poor GCA effect leading to the production of non-heterotic hybrid combinations. The positive better parent heterosis was recorded previously for various combinations in sesame (*Sesamum indicum* L.). It was observed that hybrids with significantly positive specific combining ability effect possess positive heterobeltiosis [44].

## Conclusions

A collection of 39 indigenous and exotic onion genotypes was used during current study. These genotypes were screened for nutraceutical contents by using FTIR analysis, extracted and quantified with spectroscopic /colorimetric methods. The genotypes with highest potential for pyruvic acid include onion Swat, MKS8823GO and MKS777, while 171 and 28531 showed poor concentration of Pyruvic acid content. Likewise pyruvic acid content Onion Swat and MKS8823GO also identified best for vitamin C content. The genotypes best for total flavonoids and phenolics include Onion Swat, MKS636ZU, MKS8823GO, Phulkara and MKS-TPSWP respectively. It is recommended that for further improvement of nutraceutical components in onion breeding these genotypes should be part of any crossing plan.

## Supporting information

S1 Fig FTIR spectrum representing functional groups over different wave lengths. (JPG)

S2 Fig Classification of onion genotypes into six groups based on skin color. (JPG)

S1 Table List of onion genotypes. (DOCX)

S1 Raw data Supporting information. (ZIP)

## References

[1] Pinho-Gomes AC., Knight A, Critchley J, Pennington M Addressing the low consumption of fruit and vegetables in England: a cost-effectiveness analysis of public policies. J. Epidemiol Community Health. 2021 Oct 8; 75(3):282–288. doi: 10.1136/jech-2020-214081 33070113

[2] Ülger TG, Songur AN, Çırak O, Çakıroğlu FP. Role of vegetables in human nutrition and disease prevention. vegetables—importance of quality vegetables to human health. IntechOpen London UK. 2018 Aug 22; 7–32.

[3] MehtaI. Origin and history of onions. IOSR J Humanit Soc Sci. 2017 Sep 25; 22(9):7.

[4] Ianni F, Marinozzi M, Sardella R, Natalini B. Quantitative evaluation of the pyruvic acid content in onion samples with a fully validated HPLC method. Int J Eng Res. 2015 Dec 15.

[5] Marefati N, Ghorani V, Shakeri F, Boskabady M, Kianian F, Rezaee R, Boskabady A review of anti-inflammatory, antioxidant, and immunomodulatory effects of Allium cepa and its main constituents. Pharm Biol. 2021 Feb 28; 59(1):287–302. doi: 10.1080/13880209.2021.1874028 33645419 PMC7919894

[6] Ibrahim RN, Alsalmani MS, Zedan TH. Study the antibacterial activity of aqueous extraction of onion (Allium cepa L) against Staphylococcus aureus isolated. Iraqi J Agric Sci. 2019 July. 2; 50(3):1186–1192.

[7] Rivera A, Mallor C, Garcés-Claver A, García-Ulloa A, Pomar F, Silvar C. Assessing the genetic diversity in onion (Allium cepa L.) landraces from northwest Spain and comparison with the European variability. N Z J Crop Hortic Sci. 2016 Jan 29; 44(2): 103–120.

[8] Dangi R, Kumar A, Khar A. Genetic variability, heritability, and diversity analysis studies in short day tropical onion (Allium cepa L.). Indian J Agric Sci. 2018; 88:948–57.

[9] Bureau S, Cozzolino D, Clark C J. Contributions of fourier-transform mid infrared (FT-MIR) spectroscopy to the study of fruit and vegetables: A review. Postharvest Biol Technol. 2019 Feb; 148: 1–14.

[10] Malik G, Dhatt AS, Malik AA. A review of genetic understanding and amelioration of edible allium species. Food Rev Int. 2021 Jan 5; 37(4):415–446.

[11] Assefa AD, Jeong YJ, Kim DJ, Jeon YA, Lee JR, Ko HC, et al. Assessing phenolic content and antioxidant potential diversity in Allium plants using multivariate data analysis. Hortic Environ Biotechnol. 2021 Oct 11; 59(5): 759–773.

[12] Faiza S, Hafiz NA, Zain M, Amina H, Naqshe Z, Rizwan A, et al. Role of endomycorrhizae, rhizobacteria and compost to improve phosphorus availability in onion. Asian J Agric Biol. 2020; 8:194–200.

[13] Younas M, Atiq M, Rajput NA, Abbas W, Bashir MR, Ahmad S, et al. Induction of resistance in onion against purple leaf blotch disease through chemicals. Asian J Agric Biol. 2021, 10.35495/ajab.2020.01.039.

[14] Naibaho FG, Hartanto A, Bintang M, Jamilah I, Priyani N and Putra ED. GC-MS analysis and antimicrobial activity of the aqueous extract from the bulbs of Allium chinense G. Don. cultivated in North Sumatra, Indonesia. Asian J Agric Biol. 2021(2): 10.35495/ajab.2019.12.562.

[15] Tanga M, Lewu FB, Oyedeji AO and Oyedeji OO. Yield and morphological characteristics of Burdock (Arctium lappa L.) in response to mineral fertilizer application. Asian J Agric Biol. 2020; 8(4): 511–518.

[16] Austria AB, Dulay RMR and Pambid RC. Mycochemicals, antioxidant and antidiabetic properties of Philippine sawgill mushroom Lentinus swartzii (Higher Basidiomycetes). Asian J Agric Biol. 2021: 10.35495/ajab.2020.06.365

[17] Anthon GE, Barrett DM. Modified method for the determination of pyruvic acid with dinitrophenylhydrazine in the assessment of onion pungency. J Sci Food Agric. 2003 Aug 5; 83(12):1210–1213.

[18] Cunnif P. Official methods of association of official analytical chemists. International Association Official Analytical Chemists. 2000; 2: 1–37.

[19] ZHANG SL, PENG DE NG, XU, YC, Lü, SW, WANG JJ. Quantification and analysis of anthocyanin and flavonoids compositions, and antioxidant activities in onions with three different colors. J Integr Agric. 2016 Sep 9; 15(9): 2175–2181

[20] Kaur C, Kapoor HC. Anti-oxidant activity and total phenolic content of some Asian vegetables. International J Food Sci Technol. 2002 Mar 8; 37(2): 153–161

[21] Schulz H, Baranska M. Identification and quantification of valuable plant substances by IR and Raman spectroscopy. Vib Spectrosc. 2007 Jan 16; 43(1): 13–25

[22] Clark CJ, Shaw ML, Wright KM, McCallum JA. Quantification of free sugars, fructan, pungency and sweetness indices in onion populations by FT-MIR spectroscopy. J Sci Food Agric. 2018 July 3 98(14); 5525–5533. doi: 10.1002/jsfa.9099 29687887

[23] Thampi N, Shalini JV. In vitro antibacterial studies of fresh green onion leaves and the characterization of its bioactive compounds using fourier transform infrared spectroscopy (FTIR). J Chem Pharm Res. 2015 7(3), 1757–1766

[24] Lu X, Wang J, Al-Qadiri HM, Ross CF, Powers JR, Tang J, et al. Determination of total phenolic content and antioxidant capacity of onion (Allium cepa) and shallot (Allium oschaninii) using infrared spectroscopy. Food Chemistry. 2011 Nov 15;129(2):637–44. doi: 10.1016/j.foodchem.2011.04.105 30634280

[25] Kim HY, Jackson D, Adhikari K, Riner C, Sanchez-Brambila G. Relationship between consumer acceptability and pungency-related flavor compounds of Vidalia onions. J. Food Sci. 2017 Sep 12; 82(10): 2396–2402. doi: 10.1111/1750-3841.13915 28898424

[26] Ianni F, Marinozzi M, Scorzoni S, Sardella R, Natalini B. Quantitative evaluation of the pyruvic acid content in onion samples with a fully validated high-performance liquid chromatography method. Int J Food Prop. 2016 Feb 16;19(4): 752–759

[27] Yoo KS, Pike L, Crosby K, Jones R, Leskovar D. Differences in onion pungency due to cultivars, growth environment, and bulb sizes. Scientia Horticulturae. 2006 Oct 9; 110(2): 144–149.

[28] Havey MJ, Randle W.M. Combining abilities for yield and bulb quality among long-and intermediate-day open-pollinated onion populations. J Am Soc Hortic Sci. 1996 Jan 19; 121(4): 604–608.

[29] Patil DG, Subramaniam VR. Combining ability analysis for yield and processing qualities in white onion (Allium cepa L.). Curr Agric Res J. 2020 Mar 3; 8(1): 18–24.

[30] Mlcek J, Valsikova M, Druzbikova H, Ryant P, Jurikova T, Sochor J, et al. The antioxidant capacity and macroelement content of several onion cultivars. Turk J Agric For. 2015 Dec 4;39(6): 999–1004.

[31] Chattopadhyay A, Sharangi AB, Dutta S, Das S, Denre M. Genetic relatedness between quantitative and qualitative parameters in onion (Allium cepa L.). Vegetos-An Int J Plant Res. 2013 July 9; 26(1): 151–7.

[32] Mondal S, Rahaman ST. Flavonoids: A vital resource in healthcare and medicine. Int J Pharm Pharm Pharmacol 2020 April 24, 8: 91–104.

[33] Sagar NA, Pareek S, Gonzalez-Aguilar GA. Quantification of flavonoids, total phenols and antioxidant properties of onion skin: A comparative study of fifteen Indian cultivars. J Food Sci Technol. 2020 Feb 5: 1–10. doi: 10.1007/s13197-020-04277-w 32549592 PMC7271087

[34] Patil BS, Pike LM, Hamilton BK. Changes in quercetin concentration in onion (Allium cepa L.) owing to location, growth stage and soil type. New Phytol. 1995 July; 130: 349–355.

[35] Mogren L, Gertsson U, Olsson. Effect of cultivation factors on flavonoid content in yellow onion (Allium cepa L.). XXVII International Horticultural Congres. 2006.

[36] Gökçe AF, Kaya C, Serçe S, Özgen M. Effect of scale color on the antioxidant capacity of onions. Scientia Horticulturae. 2010 Feb 2; 123(4): 431–435.

[37] Mpofu A, Sapirstein HD, Beta T. Genotype and environmental variation in phenolic content, phenolic acid composition, and antioxidant activity of hard spring wheat. J Agric Food Chem. 2006 54(4): 1265–1270. doi: 10.1021/jf052683d 16478246

[38] Anttonen MJ, Karjalainen RO. Environmental and genetic variation of phenolic compounds in red raspberry. J Food Compos Anal. 2005 Dec; 18(8): 759–769.

[39] Kaushik P. (2019). Genetic analysis for fruit phenolics content, flesh color, and browning related traits in eggplant (Solanum melongena). Int J Mol Sci. 2019 June 19; 20(12): 2990 10.3390/ijms20122990 PMC6628304 31248080

[40] Patil DG, Subramaniam VR. Combining Ability Analysis for Yield and Processing Qualities in White Onion (Allium cepa L.). Curr Agric Res J. 2020 Mar 3; 8(1).

[41] Abhishek C, Negi PS, Singh N K. Combining ability for flavonoids, flavonols and total phenols in cabbage (Brassica oleracea var. capitata L). Vegetos. 2017 30: 4. doi: 10.4172/2229, 4473, 20–39.

[42] Ahmad R, Shah MKN, Mahmood-ul-Hassan D, Javaid RA, Khan N. Assessment of genetic divergence and its utilization in hybrid development in cultivated onion (ALLIUM CEPA L.). J Anim Plant Sci. 2020 Aug 26; 31(1).

[43] Faria MV, Zaluski WL, Rosa J, Rossi ES., Resende JTV, Kobori RF, et al. (2019). Genetic divergence among inbred onion lines and correlation with heterosis and combining ability. Genet Mol Res. 2019 Aug 27; 18(3).

[44] Aladji MM, Tchiagam JB., Yanou NN, Bell JM. Genetics of Seed Phenolic Content and Antioxidant Activity in Diallel Crosses of Sesame, Sesamum Indicum L. J Biotech Res Biochem. 2018 Oct 26: IJBB–105.

