# Supplementary figures and images for: Nutraceutical profiling of elite onion germplasm and breeding hybrids with improved nutraceutical quality
Source: PLoS One. 2022 Jan 19;17(1):e0262705. doi: 10.1371/journal.pone.0262705 (PMC8769310; doi:10.1371/journal.pone.0262705)

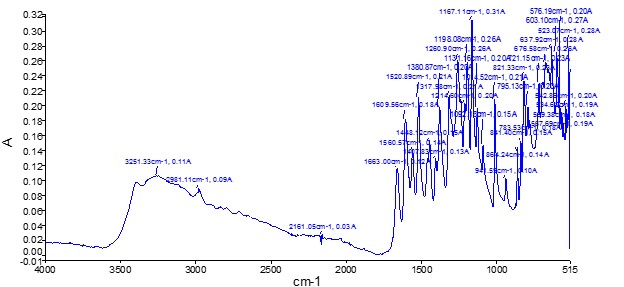

Supplement: S1 Fig — (JPG) [file pone.0262705.s001.jpg]

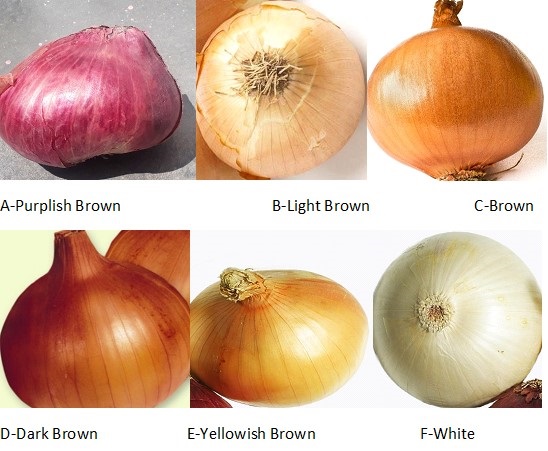

Supplement: S2 Fig — (JPG) [file pone.0262705.s002.jpg]
