# Supplementary material for: Nutraceutical profiling of elite onion germplasm and breeding hybrids with improved nutraceutical quality
Source: PLoS One. 2022 Jan 19;17(1):e0262705. doi: 10.1371/journal.pone.0262705 (PMC8769310; doi:10.1371/journal.pone.0262705)
Supplement: S1 Table — (DOCX) [file pone.0262705.s003.docx]

S1Table: List of onion genotypes

| SR.No. | Genotypes | Origin | Onion Bulb Color | Day Length |
| --- | --- | --- | --- | --- |
| 1 | 32813 | Pakistan | Light Brown | Short day |
| 2 | MKS-502 | Australia | Yellowish Brown | Short day |
| 3 | MKS-77127 | Australia | Brown | Short day |
| 4 | MKS-132807 | Australia | Brown | Short day |
| 5 | MKS-103GB | Australia | Dark Brown | Short day |
| 6 | 28533 | Pakistan | Light Brown | Short day |
| 7 | 28535 | Pakistan | Dark Brown | Short day |
| 8 | MKS-50103GW | Australia | White | Short day |
| 9 | 28529 | Pakistan | Brown | Short day |
| 10 | Super Sarhad | Pakistan | Light Brown | Short day |
| 11 | 28534 | Pakistan | Brown | Short day |
| 12 | MKS-TPSWP | Australia | Yellowish Brown | Short day |
| 13 | MKS-777 | Australia | Purplish Brown | Short day |
| 14 | MKS-636ZU | Australia | Purplish Brown | Short day |
| 15 | 28538 | Pakistan | White | Short day |
| 16 | MKS-14278 | Australia | Dark Brown | Short day |
| 17 | 28537 | Pakistan | Purplish Brown | Short day |
| 18 | Phulkara | Pakistan | Yellowish Brown | Short day |
| 19 | CGN-18750 | Pakistan | White | Short day |
| 20 | 28530 | Pakistan | Light Brown | Short day |
| 21 | MKS-5021 | Australia | Purplish Brown | Short day |
| 22 | Sand | Pakistan | Light Brown | Short day |
| 23 | CGN-16350 | Pakistan | White | Short day |
| 24 | CGN-20182 | Pakistan | Brown | Short day |
| 25 | MKS-8823GO | Australia | Purplish Brown | Short day |
| 26 | 28531 | Pakistan | Light Brown | Short day |
| 27 | MKS-1290SGB | South Africa | Yellowish Brown | Short day |
| 28 | 28532 | Pakistan | Light Brown | Short day |
| 29 | 28539 | Pakistan | Light Brown | Short day |
| 30 | 170 | Pakistan | White | Short day |
| 31 | NARC-2005 | Pakistan | Purplish Brown | Short day |
| 32 | CGN-15740 | Pakistan | Light Brown | Short day |
| 33 | CGN-24762 | Pakistan | Light Brown | Short day |
| 34 | 28540 | Pakistan | Light Brown | Short day |
| 35 | 171 | Pakistan | White | Short day |
| 36 | Onion Swat | Pakistan | Purplish Brown | Short day |
| 37 | 28536 | Pakistan | Light Brown | Short day |
| 38 | MKS-RK8138 | Australia | Light Brown | Short day |
| 39 | MKS-RDFE | Australia | Light Brown | Short day |

S1 Fig: FTIR spectrum representing functional groups over different wave lengths


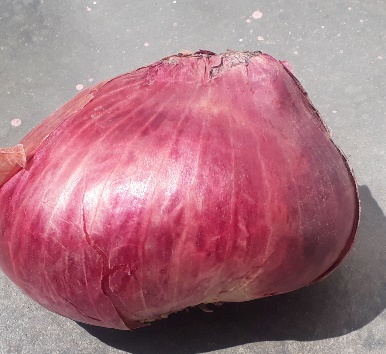

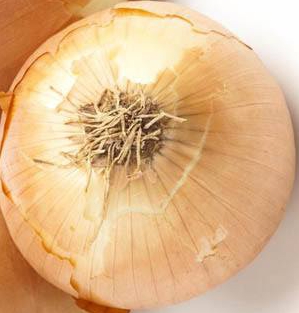

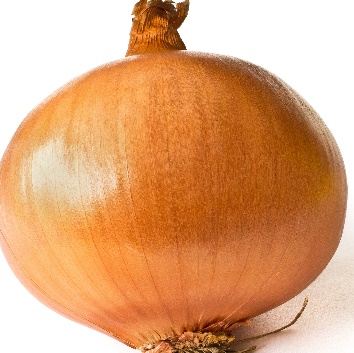


A-Purplish Brown B-Light Brown C-Brown


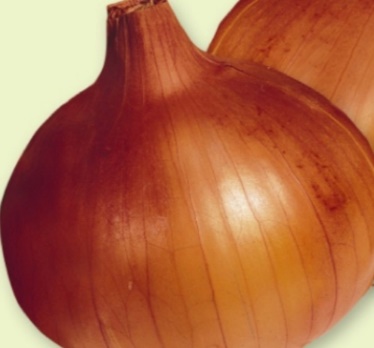

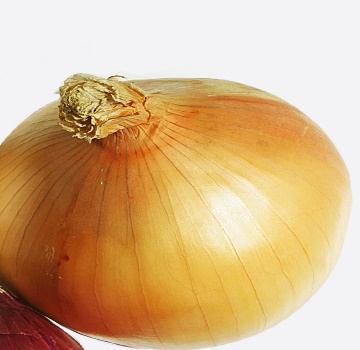

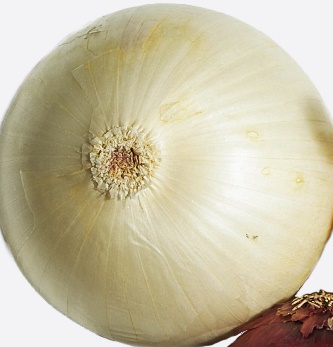


D-Dark Brown E-Yellowish Brown F-White

S2 Fig: Classification of onion Genotypes into six groups based on skin color
